# Updates, Applications and Future Directions of Deep Learning for the Images Processing in the Field of Cranio-Maxillo-Facial Surgery

**DOI:** 10.3390/bioengineering12060585

**Published:** 2025-05-29

**Authors:** Luca Michelutti, Alessandro Tel, Massimo Robiony, Lorenzo Marini, Daniele Tognetto, Edoardo Agosti, Tamara Ius, Caterina Gagliano, Marco Zeppieri

**Affiliations:** 1 Clinic of Maxillofacial Surgery, Head-Neck and NeuroScience Department, University Hospital of Udine, p.le S. Maria della Misericordia 15, 33100 Udine, Italy; micheluttiluca.uniud@gmail.com (L.M.); alessandro.tel@icloud.com (A.T.);; 2 Department of Medicine, Surgery and Health Sciences, University of Trieste, 34127 Trieste, Italy; 3 Division of Neurosurgery, Department of Medical and Surgical Specialties, Radiological Sciences and Public Health, University of Brescia, Piazza Spedali Civili 1, 25123 Brescia, Italy; 4 Academic Neurosurgery, Department of Neurosciences, University of Padova, 35121 Padova, Italy; 5 Department of Medicine and Surgery, University of Enna “Kore”, Piazza dell’Università, 94100 Enna, Italy; 6 Mediterranean Foundation “G.B. Morgagni”, 95125 Catania, Italy; 7 Department of Ophthalmology, University Hospital of Udine, 33100 Udine, Italy

**Keywords:** deep learning, cranio-maxillo-facial surgery, images processing, classification, segmentation, oral cancer

## Abstract

The entry of artificial intelligence, in particular deep learning models, into the study of medical–clinical processes is revolutionizing the way of conceiving and seeing the future of medicine, offering new and promising perspectives in patient management. These models are proving to be excellent tools for the clinician through their great potential and capacity for processing clinical data, in particular radiological images. The processing and analysis of imaging data, such as CT scans or histological images, by these algorithms offers aid to clinicians for image segmentation and classification and to surgeons in the surgical planning of a delicate and complex operation. This study aims to analyze what the most frequently used models in the segmentation and classification of medical images are, to evaluate what the applications of these algorithms in maxillo-facial surgery are, and to explore what the future perspectives of the use of artificial intelligence in the processing of radiological data are, particularly in oncological fields. Future prospects are promising. Further development of deep learning algorithms capable of analyzing image sequences, integrating multimodal data, i.e., combining information from different sources, and developing human–machine interfaces to facilitate the integration of these tools with clinical reality are expected. In conclusion, these models have proven to be versatile and potentially effective tools on different types of data, from photographs of intraoral lesions to histopathological slides via MRI scans.

## 1. Introduction

### 1.1. Background

Artificial intelligence (AI) is one of today’s most significant technological revolutions, with a particular impact on the healthcare sector. This technology allows machines to simulate typical human cognitive processes, such as learning, reasoning, and problem solving, through advanced algorithms and computational models.

**Figure 1:**
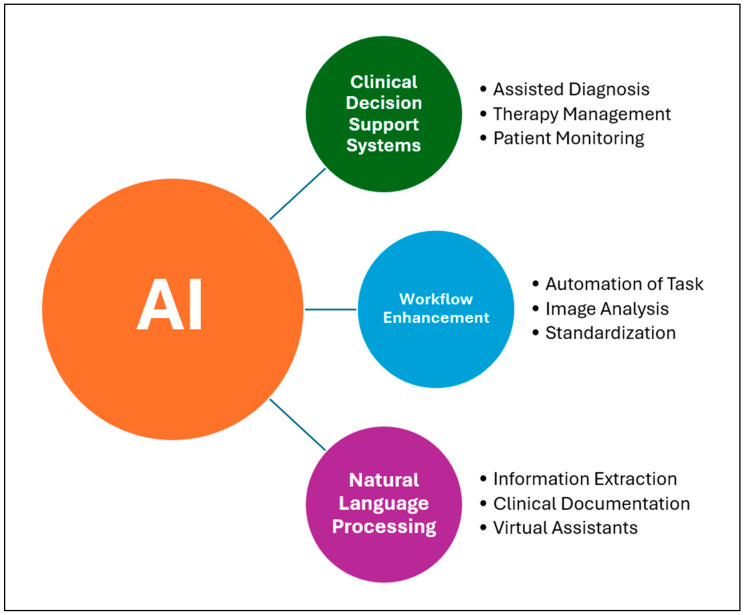
Schematic representation of main functions of AI in medicine.

In recent years, artificial intelligence algorithms have provided innovative solutions for early diagnosis, personalized treatment, resource optimization, and healthcare data management. Among the main applications of AI in medicine are Clinical Decision Support Systems (CDSSs), workflow enhancement, and Natural Language Processing (NLP). Figure 1 illustrates an overview of the classification of deep learning techniques employed in craniofacial imaging.

**Clinical Decision Support Systems** are the most promising tools based on artificial intelligence algorithms that assist physicians in decision making by analyzing clinical data, such as radiological or anatomopathological images, in order to suggest a diagnosis or treatment. Examples of applications of these supporting tools are assisted diagnosis by studying CT scans, MRI or histological examinations, therapy management by flagging drug interactions and incorrect dosages or suggesting personalized protocols, and patient monitoring [1]. A multicenter study conducted by Saha et al. (2024) [2] analyzed the performance of an AI algorithm in detecting prostate cancer, obtaining results superior to radiologists using PI-RADS and demonstrating how this system has the potential to be a supportive tool in clinical–diagnostic settings. Another interesting concept in this area is digital twins, a dynamic virtual representation of a subject, in this case the patient, using the patient’s clinical data. This tool, having limitations at present, would allow the personalization of care in the future, simulating the response to determinant drugs or treatments based on the clinical and pathological characteristics of the patient [3].

**Workflow Enhancement** is a tool that optimizes hospital workflows in various ways, such as automating repetitive processes to reduce the workload on physicians. There are AI models that improve the quality of radiological data during acquisition, for example, by denoising MRI scans. This could speed up imaging acquisition times, increasing both the availability and accessibility of care and treatment pathways [4]. Not only denoising but also segmentation, another function of some AI models, can be useful for speeding up the time of diagnosis and preoperative planning if surgery is required. The study conducted by Oh et al. (2023) [5] proposed a deep learning (DL) model for automatic segmentation of liver parenchyma and all vascular and biliary structures in MRI scans in order to optimize and improve preoperative planning. A further application of these tools is the automation of repetitive tasks as reported in the study conducted by Bizzo et al. (2021) [6]. In this study, it is shown how these models can be useful in helping clinicians perform structured reporting of radiological images of lung cancer by integrating information from multiple sources and generating structured reports to improve clinical decision support. In addition to the optimization of repetitive processes and reduction in radiological data acquisition time, as also demonstrated in the study conducted by Bharadwaj et al. (2024) [7], further functionality of these AI-based systems is the standardization of treatment protocols, homogenizing diverse practices and providing optimal care for all patients through large-scale health programs [8].

Another tool based on artificial intelligence algorithms is **Natural Language Processing** (NLP). It enables the interpretation and generation of human language. In the medical field, this function can be useful for extracting information from medical reports, analyzing unstructured clinical notes, or translating for foreign patients. This technology helps reduce the time spent on Electronic Health Record (EHR) activities, which involve documenting patient health data. AI-based virtual assistants and chatbots can help reduce the burden on physicians by assisting with medical documentation, managing tests and appointments, and activating, handling, or monitoring health programs [9]. This functionality also finds application in the field of telemedicine, for example, in the remote monitoring of chronic patients with multiple comorbidities through the use of Large Language Models, which have been tested for symptom checking, anomaly detection, and initiating care in case of deviations from usual patterns [10].

### 1.2. Machine Learning vs. Deep Learning

Artificial intelligence is a form of computer automation that enables machines to process and analyze data. Machine learning (ML) and deep learning (DL) are foundational pillars of artificial intelligence. ML, a subset of AI, is a field of study that gives computers the ability to learn without being explicitly programmed, while DL is a subfield of machine learning based on neural networks [11].

Machine learning models are simpler and more interpretable, such as decision trees or regressions, and require less data compared to deep learning, although they have lower performance on complex tasks. There are different types, including supervised learning, where data are labeled by humans, such as SVMs, Random Forests, or linear regression, and unsupervised learning, where data have no labels, such as K-means or PCA. There is also reinforcement learning, where the model learns by rewarding or penalizing its choices based on the outcomes, such as Q-Learning or Deep Q-Network.

Deep learning models are based on artificial neural networks with many layers and are particularly effective in processing complex data like images, audio, and text. DL algorithms extract complex features without human intervention, require large amounts of training data, have high computational demands, and are not easily interpretable, which is why they are called black boxes. There are various types of DL, including convolutional neural networks (CNNs) for image and video processing, recurrent neural networks (RNNs) and LSTM for sequential data like text, generative adversarial networks (GANs) for generating realistic data, and transformers, advanced NLP models used in chatbots. While deep learning (DL) is a subset of machine learning (ML), we have delineated them separately to emphasize their distinct characteristics, architectures, and applications in medical imaging. This distinction facilitates a greater comprehension of the differences between classic machine learning methods and deep learning techniques in managing high-dimensional data, especially in the realm of cranio-maxillo-facial picture analysis. The main characteristics for machine learning and deep learning are listed in Table 1.

### 1.3. Objective of This Study

The purpose of this review is to present a general overview of what the current applications of deep learning in image processing of medical data are, particularly CT and MRI scans, anatomopathological images, and clinical photos, inherent to oral cancer. An analysis of what the advantages, limitations, and future challenges of this technology are in oral surgery will be proposed.

## 2. Methods

To give this review a methodology, the PRISMA scoping review guidelines were adopted. The question we set to conduct this study is as follows: “What are the current applications of deep learning algorithms in clinical image processing in oral cancer, what are the advantages and limitations to be addressed in the future?” 

### 2.1. Literature Search

For the literature search, articles published in the last three years from 2023 to 2025 were considered, and the search was conducted until 15 April 2025. The databases that were used included the following: MEDLINE, Embase, ScienceDirect, and Cochrane central Register of Controlled Trials (CENTRAL). Studies found in the literature were imported into EndNote21 (Clarivate, Analytics, Philadelphia, PA, USA) and screened by two independent investigators (L.M.1 and A.T.). In case of doubt between the two investigators, a third independent investigator (L.M.2) was involved.

### 2.2. Inclusion and Exclusion Criteria

Primarily clinical trials and cohort studies were included. Studies to be included had to be in English, published from 2023 to 2025, and conducted on patients with oral cancer pathology. All studies that did not analyze the applicability of deep learning algorithms in processing clinical images, including photos, CT and MRI scans, and histopathological images, were included.

### 2.3. Data Collection

Data collected from the included studies comprised the following: application of the deep learning algorithm, type of algorithm and main characteristics, type of clinical data used (photos, CT/RMN scans, or histopathological images), advantages and disadvantages, limitations found in the various studies, results, and parameters used by the individual included studies.

## 3. Results

The studies included in this review all focus on the analysis of clinical, radiological, and histopathological images related to oral cancer. Following the selection process and applying the inclusion and exclusion criteria, 14 studies published between 2023 and 2025 were selected to highlight the most recent advancements in this field. Of the fourteen studies, seven analyzed clinical photographs, four focused on histological slides, two on cytological samples, and one on MRI scans.

Regarding the use of deep learning (DL) models for clinical image analysis, the study by Liyanage et al. (2023) [12] developed a DL system (DCNN—deep convolutional neural network) for the autonomous classification of oral lesions as non-neoplastic, benign, and pre-malignant or malignant. Specifically, 342 images were analyzed using two models, EfficientNetV2 and MobileNetv3, with the latter achieving an accuracy of 76% and an AUC (Area Under the Curve) of 0.88. Although the model showed potential for remote screening via smartphone devices, it reported lower accuracy for non-neoplastic lesions and raised concerns about overfitting.

The study by Chen et al. (2024) [13] analyzed 131 images and evaluated the performance of two models, CANet and Swin Transformer, for binary classification (cancer vs. non-cancer), demonstrating accuracies of 97.00% and 94.95%, respectively. Another study that used intraoral RGB images as input was conducted by Kouketsu et al. (2024) [14], aimed at detecting the position and presence of oral squamous cell carcinoma (OSCC) and intraoral leukoplakia. A DL model, the Single Shot Multibox Detector (SSD), was used to analyze 1043 images, achieving a sensitivity of 93.9% and a specificity of 81.2%.

Kusakunniran et al. (2024) [15] developed a segmentation system for tongue lesions in patients with oral cancer using an advanced CNN model, Deep Upscale U-Net (DU-UNET), which enhances the traditional U-Net architecture by adding a third up sampling stream. The analysis of 995 images demonstrated an accuracy of 99.97%, showing high precision even with complex images and outperforming the standard U-Net, although performance decreased in visually different domains.

Patel et al. (2024) [16] investigated a DL model composed of EfficientNet-B5, GAIN (Guidance Stream), and Anatomical Site Prediction (ASP) for multiclass classification of 16 types of oral lesions. Using a total of 1888 images, the model achieved an AUC of 0.99. Despite the high accuracy, its performance was lower for rarer lesion classes.

Similarly, the study by Thakuria et al. (2025) [17] developed a segmentation model for oral lesions from smartphone-captured images, aiming to make diagnosis more accessible, affordable, and non-invasive. The model, OralSegNet, is an enhanced U-Net incorporating EfficientNetV2L, ASPP, residual blocks, and SE blocks. The processing of 538 images achieved an AUC of 0.97, the highest among the models tested.

**Figure 2:**
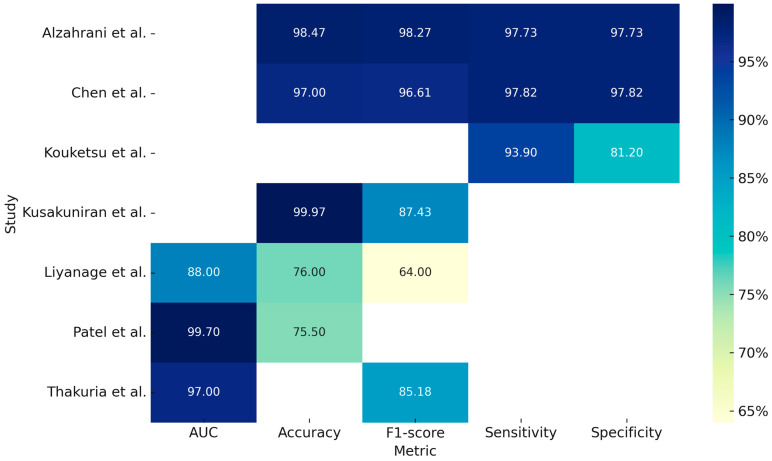
Graphical representation of measurements, expressed in percentages, obtained from the individual included studies analyzing the application of deep learning models for clinical photo processing [12,13,14,15,16,17,18].

The study by Alzahrani et al. (2025) [18] developed an integrated DL model, DSLVI-OCLSC, for the automatic classification and segmentation of oral carcinoma lesions. The model incorporates Wiener Filtering for noise reduction, ShuffleNetV2 for feature extraction, MA-CNN-BiSTM for robust classification, Unet3+ for precise segmentation, and the Sine Cosine Algorithm (SCA) for optimization. This integrated system demonstrated a high accuracy of 98.7%, despite being trained on a relatively small dataset of 131 images. Table 2 summarizes the studies included in our review based on the processing of clinical pictures and the Figure 2 is a graphical representation of measurements, expressed in percentages, obtained from the individual included studies analyzing the application of deep learning models for clinical photo processing.

In addition to clinical photographs acquired via cameras or smartphones, histological slides represent another significant data source of interest. The study conducted by Ahmad et al. (2023) [19] compared the performance of various deep learning (DL) models combined with a machine learning (ML) algorithm, specifically a Support Vector Machine (SVM), for the identification of pathological slides. By analyzing 5192 histopathological images (2494 normal vs. 2698 OSCC), the models Xception, InceptionV3, InceptionResNetV2, NASNetLarge, and DenseNet201, when combined with the ML algorithm, demonstrated an accuracy of 97% and an AUC of 0.96. Although the models showed high accuracy and reduced the workload of pathologists by enabling early diagnosis, issues of overfitting and the need for high computational resources were reported.

The study conducted by Panigrahi et al. (2023) [20] used deep convolutional neural networks (DCNNs) for automatic classification of histopathological images for the identification of OSCC, taking two approaches: the first was using networks that were already pre-trained, while the second was using a CNN model built from scratch. In this case, the ResNet50 model performed better than the others, demonstrating high accuracy and excellent generalization without overfitting.

The study conducted by Das et al. (2023) [21] also developed a CNN model from scratch, demonstrating better performance than the other models and ResNet50. Through an analysis of 1274 histopathologic images, this model demonstrated high accuracy, although it is inferred that this is not yet validated on clinical images in real time and is limited only to a binary classification (benign vs. malignant). In another study, Confer et al. (2024) [22] developed a model using a fully convolutional network (FCN) with ResNet50 for automatic, stain-free segmentation of histopathological images from potentially malignant oral biopsies (OPDM). Based on a dataset of 2561 images acquired through distal frequency infrared (DFIR) imaging, the model achieved an accuracy of 94.5%, demonstrating high precision and the feasibility of omitting histological staining.

Ragab et al. (2024) [23] introduced a novel technique named SEHDL-OSCCR for the automatic identification of OSCC from histopathological images. The model integrates SE-CapsNet for advanced feature extraction, the Improved Crayfish Optimization Algorithm (ICOA) for parameter tuning, and a CNN-BiLSTM architecture for classification. Analyzing a dataset of 528 images (439 pathological and 89 normal), the model achieved an accuracy of 98.75%.

**Figure 3:**
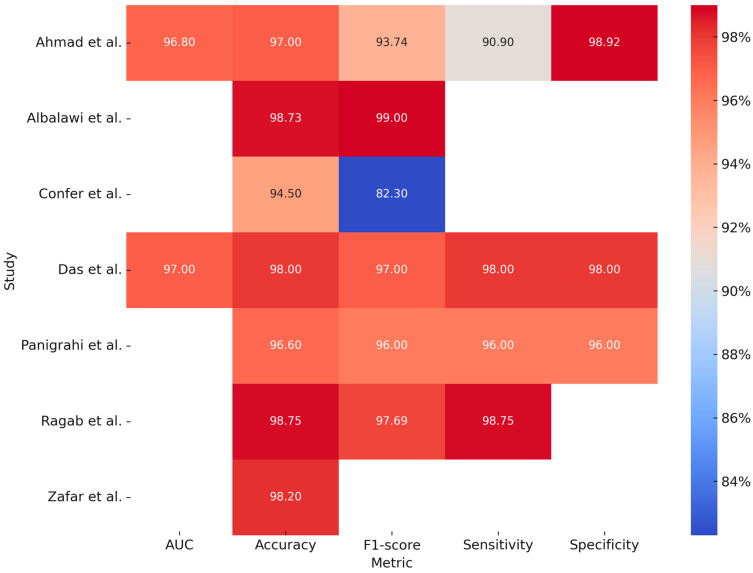
Graphical representation of measurements, expressed in percentages, obtained from the individual included studies analyzing the application of deep learning models for histopathological images processing [19,20,21,22,23,24,25].

The study by Zafar et al. (2024) [24] developed a model for the detection of OSCC using H&E-stained histopathological images. This advanced approach combined ResNet-101 and EfficientNet-b0 for feature extraction, Canonical Correlation Analysis (CCA) for feature fusion, Binary-Improved Harris Hawks Optimization (b-IHHO) for optimal feature selection, and K-Nearest Neighbors (KNN), a machine learning model, for classification. The combined model demonstrated high accuracy (98.28%) through the analysis of 4946 images (2435 normal and 2511 OSCC). Table 3 summarizes the reported studies on the processing of histological images and Figure 3 is a graphical representation of measurements, expressed in percentages, obtained from the individual included studies analyzing the application of deep learning models for histopathological images processing.

Studies have also been conducted on cytological images from specimens obtained by brushing intraoral lesions. The study conducted by Koriakina et al. (2024) [26] developed an oral cancer detection system by studying these data. Two DL models, Single Instance Learning (SIL) and Attention-Based Multiple Instance Learning (ABMIL), were compared. Analyzing the characteristics of a total of 307,839 cells, SIL showed better performance than ABMIL in terms of accuracy (93.1%).

The study conducted by Sukegawa et al. (2024) [27] constructed a classification system for oral exfoliative cytology on precancerous lesions, OSCC, and glossitis by comparing six ResNet50 templates and processing 14,535 images. Table 4 summarizes the studies included in our review about the processing of cytological images.

Regarding radiological image processing and processing, the study conducted by Yuan et al. (2023) [28] implemented a multilevel residual deep learning network (MDRL) for OSCC diagnosis on OCT (Optical Coherence Tomography) scans. By comparing the performance of the algorithm with experienced radiologists and other DL models, better performance results were obtained than those obtained by clinicians.

Another study, conducted by Chen et al. (2023) [29] analyzed the performance of a DL model fused with radiomics for the identification of cervical lymph node metastases. The DL + radiomics model compared with the two experienced clinicians showed higher sensitivity (92 vs. 72% and 60%) but lower specificity (88 vs. 97 and 99%).

**Figure 4:**
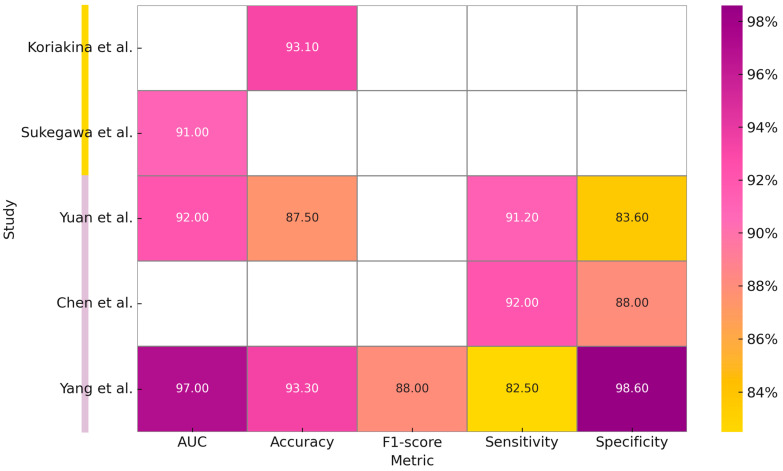
Graphical representation of measurements, expressed in percentages, obtained from the individual included studies analyzing the application of Deep Learning models for cytological images (first two studies) and CT and MRI scans processing [26,27,28,29,30].

The only study, reported in Table 5, which analyzes MRI images, is the one conducted by Yang et al. (2025) [30]. This study developed and validated a three-stage DL model for the diagnosis of cervical lymph node metastasis (LNM) in patients with oral cavity squamous cell carcinoma (OSCC), based on an AlexNet modified model and a Random Forest classifier. This model, designed to detect non-clinically visible metastases and to guide cervical dissection, demonstrated higher performance than radiologists with an AUC of 0.97 in the training set and 0.81 in the external validation. Table 5 summarizes the reported studies on the processing of MRI and CT images and Figure 4 is a graphical representation of measurements, expressed in percentages, obtained from the individual included studies analyzing the application of Deep Learning models for cytological images (first two studies) and CT and MRI scans processing.

## 4. Discussion

This study aims to provide a general overview of the most recent applications of deep learning (DL) algorithms in the processing of clinical images of patients affected by oral cancer, particularly oral squamous cell carcinoma (OSCC). The analysis of the included studies clearly demonstrates that the application of these models in the study and diagnosis of oral cancer is rapidly evolving, offering more precise and accessible tools. DL models have been employed across a wide range of clinical data, including intraoral photographs, histological slides, cytological samples, and, to a lesser extent, MRI scans.

The use of clinical photographs acquired through mobile devices such as smartphones or digital cameras represents one of the most promising applications of DL models in oncology. These approaches, although sometimes limited by overfitting, open up concrete opportunities for remote screening tools that are economically sustainable and accessible, especially in resource-limited settings. In particular, the development of smartphone applications would enable the implementation of user-friendly clinical tools, supporting the creation of large-scale, low-cost screening programs capable of facilitating early disease detection, reducing mortality rates, and improving clinical outcomes in patients with oral lesions.

In the field of pathology, the use of these tools may ease the workload of pathologists and enable not only the classification of pathological tissues but also the segmentation of histopathological images without the need for staining. This has the potential to significantly impact diagnostic workflows, reducing human labor and accelerating turnaround times. However, this technology requires high computational resources and large, well-annotated datasets. Cytological data have also shown promising results, allowing discrimination between precancerous and neoplastic lesions. The findings highlight the need for highly optimized processing techniques and careful management for large-scale cellular analysis. We have noted a significant lack of studies utilizing MRI and cytological imaging data in deep learning applications for cranio-maxillo-facial surgery. This disparity likely indicates the present availability and accessibility of annotated datasets, while also underscoring a deficiency in the literature that necessitates targeted inquiry in forthcoming studies.

Although limited to a single study, the use of DL models for the processing and analysis of MRI scans represents a highly promising frontier. This approach has shown potential in complex diagnostic tasks, such as the detection of clinically negative cervical lymph node metastases in patients with OSCC. Visual interpretation by radiologists is often subjective and may be limited by signs not visible to the human eye. DL models can provide a more objective evaluation, enhancing early detection of metastases that are clinically undetectable. This has important therapeutic implications, as it may assist clinicians in deciding whether or not to perform cervical dissection. Furthermore, these tools can reduce inter-operator variability, improve workflow efficiency, and standardize diagnostic protocols. To validate this application, large-scale multicenter studies using heterogeneous datasets are required. A key technical challenge to overcome will also be the standardization of image formats and signal quality.

Future research directions regarding the application of deep learning in clinical image analysis should focus on several key priorities. First, it is crucial to promote the development of generalizable models that can maintain high performance across heterogeneous domains. Moreover, in light of the performance limitations observed in underrepresented classes, increasing the size and diversity of training datasets is essential. This can be achieved through multicenter collaborations and the enhancement of standardized open-source databases. Another critical aspect is the multimodal integration of clinical data—combining clinical images with histopathological, anamnesis, and genomic data to enhance diagnostic accuracy and provide a comprehensive 360° view of the patient’s profile. Additionally, developing more efficient and lightweight models, tailored for mobile devices or clinical environments, represents a fundamental challenge. The spread of applications capable of analyzing intraoral lesion images may foster the development of large-scale screening programs even in remote areas. Ethical and regulatory issues must not be overlooked. It is essential to systematically address concerns such as algorithmic bias, data privacy, and medico-legal responsibility in order to enable the safe and effective clinical implementation of these technologies. Future research must emphasize the creation of multicenter annotated datasets, especially incorporating underutilized imaging modalities like MRI and cytology. Moreover, initiatives must prioritize model generalization across diverse populations and acquisition procedures, while also integrating federated learning frameworks to enable privacy-preserving collaboration among several institutions.

## 5. Conclusions

This work has highlighted the increasing use of deep learning models in clinical image processing for the diagnosis of oral cancer. These models have proven to be versatile and potentially effective tools on different types of data, from photographs of intraoral lesions to histopathological slides via MRI scans. Particularly promising is the development of applications that enable image acquisition and analysis, allowing the development of remote, inexpensive, and easily accessible screening tools. The integration of deep learning in radiology and anatomopathology also suggests an increase in diagnostic performance and an easing of workload. However, to achieve real application of such technology in clinical practice, several challenges related to generalizability of models, standardization of datasets, and legal and ethical regulation must be addressed. Only by improving and enhancing multidisciplinary approaches and large-scale validations will a safe application of deep learning in everyday reality be achieved.

## Figures and Tables

**Table 1:** Characteristics and main differences between machine learning and deep learning.

Characteristics	Machine Learning	Deep Learning
Feature Extraction	Manual	Automatic
Data required	Works well with smaller datasets	Needs large volumes of data
Hardware	Sufficient CPU	GPU/TPU required
Interpretability	More transparent and understandable models	Complex and less interpretable models
Typical applications	Regression; simple classification	Computer vision; NLP; voice recognition

**Table 2:** Schematic representation of studies based on the processing of clinical pictures.

Study	Purpose	DL Models Used	Data Type	Results	Advantages	Disadvantages
Liyanage et al. (2023) [12]	Classification of oral lesions (non-neoplastic vs. benign vs. malignant neoplastic).	EfficientNetV2 and MobileNetv3.	342 clinical photos.	Accuracy: 76% (MobileNetV3); AUC: 0.88; Precision/Recall/F1-score: ~0.64.	Usable on smartphones for remote screening; promising AUC; support in low-resource areas.	Low accuracy on non-neoplastic lesions; no texture filtering; overfitting possible.
Chen et al. (2024) [13]	Binary classification (cancer vs. healthy).	CANet and Swin Transformer.	131 clinical photos.	CANet: accuracy 97.00%; sensibility 97.82%; specificity 97.82%; F1 96.61%. Swin: accuracy 94.95%; sensibility 95.37%; specificity 95.52%; F1 94.66%.	High accuracy; robust to noise; suitable for small datasets; transferable.	Small dataset; sensitivity to low-quality images; risk of inconsistent labels.
Kouketsu et al. (2024) [14]	Detection of location and presence OSCC and leukoplakia.	Single Shot Multibox Detector (SDD).	1043 clinical photos.	Sensibility 93.9% (OSCC); 83.7% (OSCC + leukoplakia). Specificity 81.2%.	Non-invasive; inexpensive; high accuracy; suitable for remote screening or self-assessment.	Reduced performance in low light or extreme images; language only; limited to RGB images.
Kusakunniran et al. (2024) [15]	Segmentation of tongue lesions.	Deep Upscale U-Net (DU-UNET).	995 clinical photos.	Accuracy up to 99.97%; mean IoU: 93.10%; Dice: 87.43%.	High accuracy even on difficult images; outperforms U-Net and existing methods.	Performance drops on visually diverse domains; specific retraining needed.
Patel et al. (2024) [16]	Multiclass classification of 16 lesions.	EfficientNet-B5 + GAIN (Guidance stream) + Anatomical Site Prediction (ASP).	1888 clinica photos.	Balanced Accuracy: GAIN 78.7% (+7.2%); GAIN + ASP 75.5%. AUC 83.7–99.7%.	High accuracy; robustness against bias; high interpretability; generalizable.	Lower performance on rare classes; anatomical predictions sometimes increase bias (GAIN + ASP).
Thakuria et al. (2025) [17]	Segmentation of oral lesions.	OralSegNet (EfficientNetV2L + ASPP + Residual Blocks + SE Block).	538 clinical photos.	Test Dice: 0.8518; F1: 0.8518; ROC-AUC: 0.97.	High accuracy; robustness to light conditions/variations; advanced architecture; clinical validation.	More power required; future optimization needed.
Alzahrani et al. (2025) [18]	Classification and segmentation of oral carcinoma lesions.	DSLVI-OCLSC (Wiener Filterning + ShuffleNetV2 + MA-CNN-BiSTM + Unett3+ + Sine Cosine Algorithm).	131 clinical photos.	Accuracy up to 98.47%; sensitivity: 97.73%; specificity: 97.73%; F1-score: 98.27%.	High accuracy; low computational time; advanced integration between segmentation and classification.	Limited dataset; high computational demand; vulnerable to low-quality images.

**Table 3:** Schematic representation of studies on the processing of histological images.

Study	Purpose	DL Models Used	Data Type	Results	Advantages	Disadvantages
Ahmad et al. (2023) [19]	Identification of OSCC on histopathological images using a combination of DL and ML models and traditional features.	Three strategies: 1. Transfer learning (based on 5 models: Xception, InceptionV3, InceptionResNetV2, and NASNetLarge e DenseNet201); 2. Hybrid CNN + SVM (deep features extracted from each CNN and classified with SVM); 3. Fusion of CNN + traditional features (texture, shape, and local texture).	5192 histopathologic images.	The combined model obtained an accuracy of 97.00%, precision of 96.77%, sensibility of 90.90%, specificity of 98.92%, F1-score of 93.74%, and AUC of 96.80%.	High accuracy; reduced burden on pathologists; early diagnosis; easily adaptable to images from different sources; fast turnaround time.	High computational cost; binary dataset (normal vs. OSCC); lack of multicenter external validation.
Panigrahi et al. (2023) [20]	Automatic classification of histopathological images to detect presence of OSCC with deep convolutional neural networks (DCNNs).	Two approaches. 1. Transfer learning on pre-trained models (Re-sNet50; InceptionV3; MobileNet; VGG16; and VGG19); 2. CNN baseline model (co-built from scratch with 10 convolutional layers).	4000 histopathologic images.	ResNet50 demonstrates higher accuracy; F-score precision; and recall than the other models (96.6%; 0.97; 0.96; and 0.96; respectively).	High accuracy with few training epochs; superior results compared with CNN model developed from scratch; excellent generalization without overfitting (due to normalization; dropout; and data augmentation techniques).	Need much time for training of complex models (such as VGG19); histological images differ greatly from ImageNet images.
Das et al. (2023) [21]	Automatic classification of histopathological images of OSCC.	Model developed from scratch: customized 10-layer CNN (8 convolutional layers, 6 max pooling layers + 6 batch normalization layers, 1 dropout layer, 2 fully connected layeers, and an output layer) compared with other DL models.	1224 histopathologic images.	The presented model demonstrated higher accuracy, precision, recal, specificity, F1-score, and AUC than the other models and ResNet50 (98%, 0.97, 0.98, 0.97, 0.97, and 0.97, respectively).	High accuracy; robust generalization; includes batch normalization and dropout; requires no manual feature extraction.	Dataset initially unbalanced and limited (need for aug-mentation); not yet validated on real-time clinical images; applicability only by binary classification (benign vs. malignant).
Confer et al. (2024) [22]	Histopathological diagnosis without staining (label-free) using discrete IR spectroscopic imaging (DFIR). Segmentation into connective tissue; dysplastic and non-dysplastic epithelium.	Fully convolutional network (FCN) on ResNet50 backbone.	2561 histopathological images	DFIR + darkfiled: accuracy 94.5%; F1-score 0.823.	Rapid workflow; high precision; avoids coloring; lower cost; high accuracy and F1-score; high clinical scale-bility.	Limited sample variability; no cancerous tissues included; multi-center validation needed.
Ragab et al. (2024) [23]	Identification of OSCC from histopathological images.	Advanced hybrid pipeline: 1. Preprocessing (bilateral filtering; BF); 2. Feature extraction (SE-CapsNet); 3. Optimization (Improved Crayfish Optimization Algorithm; ICOA); 4. Final classification (CNN + BiLSTM).	528 histopathological images	Compared with other recent DL models; such as ResNet50 or VGG16; the SEHDL-OSCCR model proposed by the study per-formed as follows: accuracy—98.75%; precision—96.69%; recall—98.75%; F1—97.69%.	High accuracy; computational efficiency (2.70 s); advanced model combination; high generalization with minimal overfitting.	Possible scalability issues; need for more validation; sensitivity to subtle variations; clinical interpretability of complex networks (CapsNet; BiLSTM) still limited.
Zafar et al. (2024) [24]	Automated framework for diagnosis of OSCC from histopathological images using a combination of DL patterns.	Combination of models: 1. Feature extractor (ResNet-101 + EfficientNet-b0 + other 14 models); 2. Feature fusion (Canonical Correlation Analysis; CCA); 3. Feature selection (Binary-Improved Harris Hawks Optimization; b-IHHO); 4. Classifier (K-Nearest Neighbors; KNN).	4946 histopathological images	The proposed model (b-IHHO + KNN) achieved an accuracy of 98.20%; higher than ResNet101 and EfficientNetb0 considered alone.	High accuracy; efficient size reduction; high generalizability.	Limited and binary dataset; non-end-to-end approach (requires multiple steps such as extraction, fusion, selection and classification); limited validation.
Albalawi et al. (2024) [25]	Automated model for detecting OSCC on histopa-tological images.	EfficientNetB3	1224 histopathological images	The model in the testing set demonstrated an accuracy of 98.73% with an F1-score of 0.99.	High accuracy; high performance on images with different resolutions (100× and 400×); robust generalization; potential clinical integration.	Potential biases related to dataset origin; uncertain generalization; poorly interpretable model (DL decisions are “black-box”); need for high computational resources.

**Table 4:** Schematic representation of studies based on the processing of cytological images.

Study	Purpose	DL Models Used	Data Type	Results	Advantages	Disadvantages
Koriakina et al. (2024) [26]	Oral cancer detection on cytological images.	Two approaches: Single Instance Learning (SIL) and Attention-Based Multiple Instance Learning (ABMIL).	Cytological images (24 patients and 307,839 cells).	Accuracy 93.1% (SIL, ResNet18); precision SIL > ABMIL on all datasets.	SIL is simpler and more accurate than ABMIL; it is high accuracy, interpretable, robust, and less prone to overfitting on irrelevant features.	ABMIL is complex, memory-heavy, less effective on small datasets and sparse key instances, and assumes that all patient cells share a label (this introduces noise).
Sukegawa et al. (2024) [27]	Classification of precancerous lesions, OSCC, and glossitis.	ResNet50 pretrained on ImageNet. Made 6 variants of the DL model (patho-logist A, B, C, and GT, majority voting, and probabilistic model).	14,535 images.	Probabilistic model has better AUC (0.91).	High accuracy; use of diversified labels; robust to single annotator bias; generalizable.	Costly labeling; variability among pathologists; retrospective and single-center study; performance not statistically comparable across dataset.

**Table 5:** Schematic representation of studies based on the analysis of MRI and CT images.

Study	Purpose	DL Models Used	Data Type	Results	Advantages	Disadvantages
Yuan et al. (2023) [28]	Diagnosis of OSCC with MDRL System compared with radiologist and other CNN models.	Multi-level residual deep learning network (MDRL).	468 OCT scans.	A sensitivity of 91.2%, specificity of 83.6%, accuracy of 87.5%, VPP of 85.3%, and VPN of 90.2% at the image level, with an AUC value of 0.92, compared to the radiologist (100, 69.3, 86.2, 80, and 100).	Non-invasive; integrates multi-level features (improving diagnostic accuracy).	Need annotated data; prefe-ribly with se-mi-supervised or non-supervised learning; model is based on 2D slicing of 3D images.
Chen et al. (2023) [29]	DL and radiomics model to identify cervical lymph node metastasis in patients with OSCC though analysis of CT scans.	3DDenseNet modifiedfor volumetric analysis of lymph nodes on CECT images (adjunctive module discrimintive filter learning; DFL).	CECT scans of 100 patients and 217 meta-static and 1973 nonmetastatic cervical lymph nodes.	The DL + radiomics model compared to the two expert clinicians demostrated higher sensiblity (92 vs. 72% and 60%) but lower specificity (88 vs. 97 and 99%).	Greater sensibility than clinicians; radiomic features and DL complementary integration; good genera-lizability.	Minor specificity to clinicians; single-center and retrospective study; model is still suboptimal for small lymph nodes or those with inconspicuous features.
Yang et al. (2025) [30]	Diagnosis of cervical lymph node metastasis in patients with OSCC cN0.	Three stages model (images-based, sequence-based, and patient-based stages). AlexNet modified + RF classifier.	45,664 MRI scans (723 patients).	The model showed better performance, with an AUC of 0.70 and an accuracy of 73.53% compared to radiologists (accuracy of 61.76%). Model performance included AUC: 0.97 (train), 0.81 (external test); ACC: 93.3%; specificity: 98.6%; sensitivity: 82.5%; and F1 score: 0.88 (train).	Reduction in occult metastasis; superior performance to radiologists; multicenter validation.	Complex model; requires high-quality MRI; retrospective study; only imaging as input.

## Data Availability

The data are available upon request.
